# Grand challenges in entomology: Priorities for action in the coming decades

**DOI:** 10.1111/icad.12637

**Published:** 2023-03-20

**Authors:** Sarah H. Luke, Helen E. Roy, Chris D. Thomas, Luke A. N. Tilley, Simon Ward, Allan Watt, Manuela Carnaghi, Coline C. Jaworski, Maximillian P. T. G. Tercel, Charlie Woodrow, Susmita Aown, Jennifer A. Banfield‐Zanin, Sarah L. Barnsley, Iris Berger, Mark J. F. Brown, James C. Bull, Heather Campbell, Ruth A. B. Carter, Magda Charalambous, Lorna J. Cole, Martin J. Ebejer, Rachel A. Farrow, Rajendra S. Fartyal, Miriam Grace, Fiona Highet, Jane K. Hill, Amelia S. C. Hood, Eleanor S. Kent, Frank‐Thorsten Krell, Simon R. Leather, Daniel J. Leybourne, Nick A. Littlewood, Ashley Lyons, Graham Matthews, Louise Mc Namara, Rosa Menéndez, Peter Merrett, Sajidha Mohammed, Archie K. Murchie, Michael Noble, Maria‐Rosa Paiva, Michael J. Pannell, Chooi‐Khim Phon, Gordon Port, Charlotte Powell, Stewart Rosell, Francisca Sconce, Chris R. Shortall, Eleanor M. Slade, Jamie P. Sutherland, Jamie C. Weir, Christopher D. Williams, Natalia B. Zielonka, Lynn V. Dicks

**Affiliations:** ^1^ School of Biosciences University of Nottingham, Sutton Bonington Campus Nr Loughborough UK; ^2^ Department of Zoology University of Cambridge Cambridge UK; ^3^ UK Centre for Ecology and Hydrology, MacLean Building Crowmarsh Gifford, Wallingford UK; ^4^ Leverhulme Centre for Anthropocene Biodiversity, Department of Biology University of York York UK; ^5^ Royal Entomological Society, The Mansion House St Albans UK; ^6^ UK Centre for Ecology & Hydrology Bush Estate Midlothian UK; ^7^ Department of Agriculture Health and Environment, Natural Resources Institute University of Greenwich at Medway Kent UK; ^8^ Cardiff University School of Biological Sciences Cardiff UK; ^9^ University of Lincoln, School of Life and Environmental Sciences Joseph Banks Laboratories Lincoln UK; ^10^ University of Sussex Falmer, Brighton UK; ^11^ Stockbridge Technology Centre Cawood, Selby, North Yorkshire UK; ^12^ School of Biological Sciences University of East Anglia Norwich UK; ^13^ Centre for Ecology, Evolution and Behaviour, Department of Biological Sciences, School of Life Sciences and the Environment Royal Holloway University of London Egham UK; ^14^ Swansea University, Singleton Park Sketty, Swansea UK; ^15^ Agriculture and Environment Department Harper Adams University Newport UK; ^16^ Lancaster Environment Centre Lancaster University Lancaster UK; ^17^ Department of Life Sciences Imperial College London, South Kensington Campus London UK; ^18^ Integrated Land Management SRUC, Auchincruive Estate Ayr UK; ^19^ Amgueddfa Cymru — National Museum Wales Cardiff UK; ^20^ University of Lincoln Brayford Way Lincoln UK; ^21^ Department of Zoology, Birla Campus HNB Gahrwal Univeristy Srinagar Garhwal Uttarakhand India; ^22^ SASA (Science and Advice for Scottish Agriculture) Edinburgh UK; ^23^ University of York, Leverhulme Centre for Anthropocene Biodiversity & Department of Biology University of York York UK; ^24^ Centre for Agri‐Environmental Research, School of Agriculture, Policy and Development University of Reading Reading UK; ^25^ Department of Zoology Denver Museum of Nature & Science Denver Colorado USA; ^26^ Zoological Biodiversity, Institute of Geobotany Leibniz University Hannover Hannover Germany; ^27^ SRUC (Scotland's Rural College) Craibstone Estate Bucksburn, Aberdeen UK; ^28^ RSPB Centre for Conservation Science Haweswater, Naddle Farm, Bampton Cumbria UK; ^29^ Imperial College, Silwood Park Ascot UK; ^30^ Teagasc, Crop Science Department, Oak Park Crops Research Centre Carlow Ireland; ^31^ 5 Castle St Essex UK; ^32^ Department of Zoology M.E.S Mampad College Mampad, Malappuram Kerala India; ^33^ Agri‐Food & Biosciences Institute Newforge Lane Belfast, Northern Ireland UK; ^34^ Steward's Cottage Hall Farm Stalham, Norfolk UK; ^35^ CENSE ‐ Center for Environmental and Sustainability Research, NOVA School of Science and Technology NOVA University Lisbon Caparica Portugal; ^36^ The Essex Field Club Colchester, Essex UK; ^37^ Entomology Branch Forest Research Institute Malaysia (FRIM) Kepong Selangor Malaysia; ^38^ Newcastle University, School of Natural and Environmental Sciences Newcastle University Newcastle upon Tyne UK; ^39^ Ulster University Belfast UK; ^40^ Rothamsted Research West Common Harpenden UK; ^41^ Asian School of the Environment Nanyang Technological University Singapore; ^42^ Eurofins Agroscience Services Ltd. Wilson, Melbourne, Derbyshire UK; ^43^ Institute for Evolutionary Biology University of Edinburgh Ashworth Laboratories Edinburgh UK; ^44^ School of Biological and Environmental Sciences Liverpool John Moores University Liverpool UK

**Keywords:** climate change, conservation, disease vector, ecosystem services, education, funding and research priorities, insect biodiversity, insect taxonomy, land use, pest control

## Abstract

1. Entomology is key to understanding terrestrial and freshwater ecosystems at a time of unprecedented anthropogenic environmental change and offers substantial untapped potential to benefit humanity in a variety of ways, from improving agricultural practices to managing vector‐borne diseases and inspiring technological advances.
2. We identified high priority challenges for entomology using an inclusive, open, and democratic four‐stage prioritisation approach, conducted among the membership and affiliates (hereafter ‘members’) of the UK‐based Royal Entomological Society (RES).
3. A list of 710 challenges was gathered from 189 RES members. Thematic analysis was used to group suggestions, followed by an online vote to determine initial priorities, which were subsequently ranked during an online workshop involving 37 participants.
4. The outcome was a set of 61 priority challenges within four groupings of related themes: (i) ‘Fundamental Research’ (themes: Taxonomy, ‘Blue Skies’ [defined as research ideas without immediate practical application], Methods and Techniques); (ii) ‘Anthropogenic Impacts and Conservation’ (themes: Anthropogenic Impacts, Conservation Options); (iii) ‘Uses, Ecosystem Services and Disservices’ (themes: Ecosystem Benefits, Technology and Resources [use of insects as a resource, or as inspiration], Pests); (iv) ‘Collaboration, Engagement and Training’ (themes: Knowledge Access, Training and Collaboration, Societal Engagement).
5. Priority challenges encompass research questions, funding objectives, new technologies, and priorities for outreach and engagement. Examples include training taxonomists, establishing a global network of insect monitoring sites, understanding the extent of insect declines, exploring roles of cultivated insects in food supply chains, and connecting professional with amateur entomologists. Responses to different challenges could be led by amateur and professional entomologists, at all career stages.
6. Overall, the challenges provide a diverse array of options to inspire and initiate entomological activities and reveal the potential of entomology to contribute to addressing global challenges related to human health and well‐being, and environmental change.

## Graphical abstract

- We present a set of grand challenges for entomology in the 21st Century, identified by members of the UK's Royal Entomological Society (RES), using a participatory prioritisation approach.
- The 61 priority challenges, falling into 11 themes, provide a diverse array of options to inspire and initiate entomological activities, including research, funding, technology development, and outreach and engagement.
- Together, they represent a bright future for a science of entomology that deliver benefits to humanity and safeguards insect biodiversity.

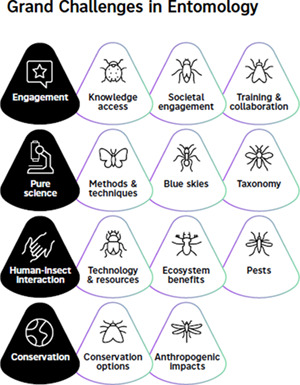

## INTRODUCTION

Insects are the most diverse animal group within terrestrial ecosystems, with about 1 million species currently described, and the total number of species estimated to be around 5.5 million (Stork, 2018). As well as being diverse, they are also very abundant and play critical roles in ecosystems, including as predators, prey, decomposers, and pollinators (Losey & Vaughan, 2006; Wilson, 1987). Several of these functional roles provide crucial ecosystem services to humans, including aiding removal of waste materials such as carrion and dung, contributing to nutrient cycling and soil processing, and pollinating 75% of the world's major food crops (Klein et al., 2007), including those plants responsible for >90% of vitamin C available for human nutrition (Eilers et al., 2011). Insects can provide us directly with food or be used as food for livestock (van Huis, 2013) and have played a valuable role in the development of life saving medicines such as antimicrobial and anticancer agents (Medeiros Costa‐Neto, 2005). They have inspired technological innovations, including advances in robotics, adhesives, and optics (Gorb, 2011). However, in addition to this wide range of positive contributions to human society, insects are also pests and vectors for disease. Arthropods—of which insects are the major component—are estimated to destroy between 18% and 26% of agricultural crop production annually across the world (Culliney, 2014; Sharma et al., 2017), whilst some insect groups cause substantial damage to forests (Bentz et al., 2019), wooden infrastructure (Govorushko, 2019), furnishings and clothing (Plarre & Krüger‐Carstensen, 2011). It is estimated that 17% of infectious diseases in humans are vector‐borne, and many of these including dengue, typhus, tick‐borne encephalitis, and sleeping sickness, are transmitted by insect and allied vectors (World Health Organization, 2020a). Malaria alone—spread by *Anopheles* mosquitoes—caused an estimated 229 million cases and 409,000 deaths in 2019 (World Health Organization, 2020b) and is one of the leading causes of death of children under the age of five in sub‐Saharan Africa (World Health Organization, 2020a).

Understanding, supporting, and responding to the myriad roles that insects play in ecosystems, and the services and disservices that they cause for humans, demands well‐developed scientific knowledge of the taxon. Entomology is the scientific discipline and branch of natural history that seeks to understand the ecology, physiology, distribution, and classification of insects. It includes a broad range of topics, including medical and veterinary entomology, pest control, and insect ecology and conservation, and has been facilitated by key scientific developments such as the invention of the microscope, and the Linnaean classification system (Leather, 2015; Smith & Kennedy, 2009). In recent decades, molecular techniques have provided further opportunities for understanding insects (e.g. DNA barcoding; Jinbo et al., 2011), and new techniques capable of further transforming entomology are constantly emerging (e.g. deep learning and computer vision; Høye et al., 2021). In the 21st century, the rapid pace of anthropogenic change of ecosystems, global challenges such as climate change and widespread biodiversity loss (Díaz et al., 2019; Newbold et al., 2016; Wagner et al., 2021), and the continued emergence of new pests and invasive non‐native species (Pyšek et al., 2020), all highlight the importance of further developing our understanding of insects, to maximise the benefits and minimise harm associated with them (Leather, 2015). We also need to continue our exploration of fundamental questions about life on Earth.

Entomological societies around the world are questioning the role that they, and their discipline, can play in developing strategies for the coming decades, including what entomologists can do for humanity, what entomology can achieve, and what directions the discipline could, and should, take next. To this end, the Entomological Society of America (ESA) initiated the ‘Grand Challenges in Entomology’ Project in 2017—a global initiative to develop ‘An entomology agenda to improve the human condition’ (https://entomologychallenges.org/). The ESA's focus was on resolving insect‐related problems or using insects to develop solutions to the ‘grand challenges’ humans will face in coming decades. Their priorities were decided by the society's board members and concentrated on three overarching strategic challenges—Public Health, Feed the World, and Invasive Species. As a response to the ‘Grand Challenges’ initiative, The Royal Entomological Society (RES)—a UK‐based entomological society, comprising 1598 members from around the world, and from a wide range of professional backgrounds—began its own complementary ‘Grand Challenges in Entomology’ programme to contribute ideas, using an alternative approach based on broad consultation of the membership, and inclusion of a wide range of topics. The aim was to generate a list of specific ideas for action, which would sit alongside the ESA's list, and those of other organisations contributing to the initiative, to provide a range of options and perspectives, to help develop an entomological agenda for the 21st century.

Through the ‘Grand Challenges’ programme, the RES plans to develop a range of ideas to inspire and direct future work around the world. The first stage, reported here, has been to engage with a wide range of entomologists with differing specialities and interests, drawn from the RES's membership and others involved in its activities. The RES's desire for inclusivity and to draw on this breadth of expertise meant that a participatory, or collaborative methodology was appropriate (sensu *lato* Sutherland et al., 2011), based on principles of openness and democracy, which aimed to gather a broad set of opinions from different perspectives.

Collaborative exercises to set research priorities bring together multiple stakeholder or informed groups to identify priority questions or information needs for new research, engagement, or activities. They are useful for aligning research with policy and practice and for developing consensus among researchers and practitioners (Dey et al., 2020; Rudd, 2011). Such exercises typically include ‘solicitation of questions and priorities from an extensive community, online collation of material, repeated voting and engagement with policy networks to foster uptake and application of the results’ (Sutherland et al., 2011). The exact format of each exercise is case dependent and can be adjusted according to the aims, community, and resources available. A set of 41 examples from ecology, biodiversity and environmental science were reviewed in detail by Dey et al. (2020). They have been used successfully in many environmental science or policy contexts (Dicks, Bardgett, et al., 2013; Sutherland et al., 2021; zu Ermgassen et al., 2020), including to identify key knowledge needs for the conservation of wild insect pollinators (Dicks, Abrahams, et al., 2013).

In this article, we describe the collaborative exercise that was conducted by the RES to identify a range of current and future ‘Grand Challenges in Entomology’. We explain the methods used to achieve this and present the key themes and final list of priority challenges that emerged from the exercise. We also begin to consider what this means for the direction that entomology should take, what entomologists can do for society, and what entomology can achieve in the coming decades.

## METHODS

**FIGURE 1:**
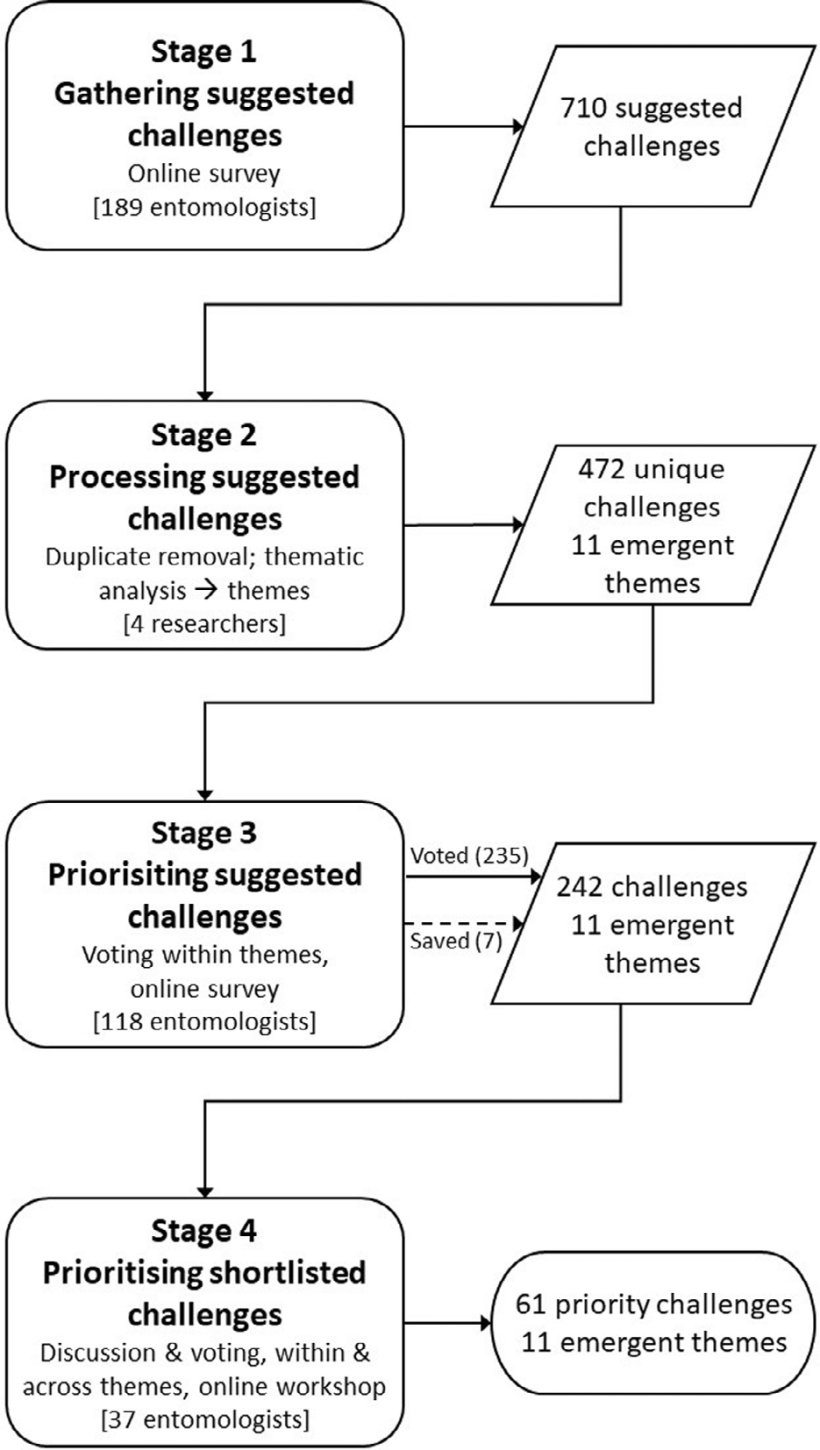
Flowchart representation of the collaborative prioritisation exercise. Boxes on the left describe stages of the process. Square brackets show how many people were involved in prioritisation steps at each stage; only scorers are counted in Stage 4, not including the steering group, facilitators and scribes. Boxes on the right show the outputs of each stage. At Stage 3, most suggested challenges (numbers in round brackets) were voted for by participants in the online survey; seven were saved subsequently as ‘wildcards’ (see text), despite receiving no online votes. For further details of Stage 4, see Figure S1.

We followed a structured collaborative process with four stages (Figure 1, Appendix S1, and Figure S1). The key aspects of each stage and subsequent data analysis and visualisation are outlined below, with additional method details in the Appendix S1.

### Stage 1: Gathering suggested challenges

We invited all RES members and fellows, including journal editorial teams and special interest group members (1598 people, from across 51 different countries—hereafter referred to collectively as ‘members’) to submit suggestions for Grand Challenges, which were defined as ‘Priority topics on which you think entomologists should focus their efforts over the coming years and decades’. We asked them to consider how they saw the future of entomology, what they thought entomologists should be concentrating their efforts on, and also what entomology could achieve, and to suggest challenges specific enough for a programme of activities or research to be designed around. We limited the suggestion length to 280 characters, and each member was allowed to submit up to five ideas. Participants were asked a series of demographic questions, comprising details about their involvement with the RES, their gender, age, country of residence, main current category of entomological activity (e.g. university academic, private sector, policy maker, amateur entomologist, etc), RES journal preferences, and years of experience as an entomologist. Data were collected between 29 October and 20 November 2020 using the online survey software Qualtrics (Qualtrics, Provo, UT). Full wording of the Stage 1 online survey is available in Appendix S2.

Before moving to Stage 2, demographic data of respondents were analysed (see Section Data analysis and visualisation) to check that a representative subset of the members had been surveyed. The results (see Section Results) were discussed by the steering group and were considered to be representative, with no need for further targeted action to increase responses from under‐represented groups.

### Stage 2: Processing suggested challenges

Four members of the research team (S.H.L., M.C., M.P.T.G.T., and C.W.) independently read the full list of suggested challenges and manually developed a thematic framework for grouping them. The same four researchers independently sorted successive subsets of 50 of the suggested challenges, allocating each to a theme within the agreed framework. Agreement in how the challenges were sorted into themes was assessed using Kappa analyses (see Appendix S1 for details). Once the researchers were sorting with sufficient consistency, the remaining 610 suggestions were sorted by a single team member (S.H.L.). Duplicate suggestions were then amalgamated by S.H.L. to avoid repetition within the list of suggestions (Fleiss et al., 2004; Gamer et al., 2019).

In a final processing step (carried out by two of the authors: A.W. and S.H.L.), some suggested challenges relevant to more than one theme were moved to the theme containing fewest suggestions to reduce variability in the number of suggested challenges per theme. One theme (‘Insect declines and conservation’) was split into its two component parts, to even out theme sizes for Stages 3 and 4.

### Stage 3: Prioritising suggested challenges

The 1256 RES members on the RES mailing list (including non‐respondents at Stage 1, excluding journal editorial teams and special interest group members) were invited to participate in a second online Qualtrics survey, run between 24 June and 8 July 2021, to begin prioritising suggested challenges.

Each participant prioritised suggested challenges from two of the themes from Stage 2: one which they felt they had expertise in, and a second that was randomly assigned, to ensure good coverage of responses across themes.

In each theme, participants were asked to read between 29 and 60 suggested challenges (depending on length of the suggestions list within each theme), presented in a randomised order, and to select the highest priority 10% from the set. Suggested challenges amalgamated from duplicates were indicated, and participants could access the original suggestions for these, to see where they came from. Free text boxes allowed participants to add comments on each challenge.

The survey included a set of demographic questions to assess the diversity of responses (as in Stage 1), and a question about willingness to participate in an online workshop, with specified dates, to prioritise the shortlisted challenges (Stage 4).

### Stage 4: Prioritising shortlisted challenges

The final prioritisation took place during an online workshop conducted on 21 and 22 July 2021, using the video communications software Zoom (see Figure S1 and Table S1 for further details). Before the workshop, collated results from Stage 3 were shared with participants in spreadsheet form, with voter identities anonymised. The challenges that received the most votes within each theme (see Appendix S1, Table 1, Table S2) were proposed for discussion in the workshop, but each participant had the opportunity to reinstate low voted ‘wildcards’ for discussion, by contacting the organisers in advance of the workshop and providing a justification. Participants were asked to prepare to introduce between two and four of the top‐voted suggested challenges during the workshop, to open discussions about each suggestion.

**TABLE 1:** Final edited text of the selected priority challenges within each theme.

Theme	Priority challenge for entomology	Day 1 results: Within‐theme rank	Day 2 results: Cross‐theme rank
Taxonomy	**Training for taxonomists**: increase resources from Government and funding agencies for training in taxonomy, particularly in tropical regions	1	AQ
	**Funding for taxonomy:** increase funding to support taxonomy and species descriptions, especially in regions with large proportions of undescribed fauna	2	AQ
	**Early career development:** provide opportunities for the early career development of taxonomists, including grants to support museum conservators	3	AQ
	**Molecular and classical taxonomy**: integrate molecular and classical taxonomy in research and education	6	6
	**Communication:** communicate the role of specimen collection and curation in entomology, to encourage a new generation to take up insect taxonomy, both professionally and at an expert amateur level	5	14
	**Museum collections**: support the digitisation of museum entomology collections	4	20
Blue Skies	**Ecological networks:** research the multiple ways insects interact and how their networks underpin biodiversity across the world	1	AQ
	**Ecological functions:** assess ecological functions in entomology	2	AQ
	**Life‐history research:** support life‐history research to underpin ecology	4	3
	**Funding**: increase funding available for curiosity‐driven—‘blue skies’—research on insects	3	17
	**Pollinator interactions:** research the interactions between wild insect pollinators and wild plants	5	24
Methods and Techniques	**Global monitoring of insects:** establish a global network of insect monitoring sites that allow long‐term monitoring of insect diversity and abundance over space and time	1	AQ
	**Identification technologies for non‐experts:** develop technologies, such as automated ID, to facilitate insect identification by non‐experts, including in citizen science projects and agriculture	2	AQ
	**Novel monitoring techniques:** develop new and effective biodiversity monitoring techniques for poorly recorded insect groups, so changes in abundance and status can be measured reliably	4	12
	**Insect genetics:** enhance the use of genetic methods to increase knowledge about the impacts of environmental change on insects	3	21
Anthropogenic Impacts	**Global declines:** evaluate whether insect declines are global in extent	1	AQ
	**Causes of change:** identify the main drivers of insect change and their relative importance in different biomes	2	AQ
	**Consequences of change:** evaluate the ecological consequences of losses and/or changes to insect diversity	3	AQ
	**Insect resilience to environmental change:** evaluate how quickly/completely insects can respond to changes, including in vulnerable ecosystems such as peatlands	Joint 5	2
	**Climate change impacts**: quantify the impacts of climate change on insect dispersal, migration, behaviour and interactions	Joint 5	4
	**Tipping points:** increase understanding of the role of tipping points and non‐linearities in the effects of change in insect communities on ecosystems	4	5
Conservation Options	**Agricultural landscape management:** evaluate how agricultural landscapes can be managed to promote insect diversity and reverse insect declines, while also providing food security	1	AQ
	**Corridors:** assess the effectiveness of riparian, hedgerow, and urban corridors in facilitating insect movement, dispersal and long‐term persistence	2	AQ
	**Rewilding impacts:** understand the impacts of vertebrate and vegetation rewilding projects on invertebrates, compared to other conservation initiatives	3	AQ
	**Urban conservation:** develop insect conservation strategies for urban areas, including ‘retro‐fitting’ cities for insects, urban‐greening and rewilding, and strategies for new housing developments.	4	AQ
	**Role of natural habitat protection:** evaluate the potential for international policies that aim to protect large areas of natural or semi‐natural habitat (e.g. ‘30 by 30′; Dinerstein et al., 2019) to reverse observed insect declines	5	1
	**Landscape‐scale conservation:** consider insects in landscape‐scale conservation planning and projects	6	22
Ecosystem Benefits	**Insects' contributions to people**: communicate and inform about the many different contributions that insects make to human well‐being, for example through ecosystem services	1	AQ
	**Understudied taxa:** increase public understanding of understudied insect taxa (e.g. parasitic wasps and flies), their ecosystem functions and the benefits they provide to people and nature	2	AQ
	**Soil biodiversity:** research the role of biodiversity in soil health/quality, including food webs, species interactions and interdependencies.	Joint 3	AQ
	**Impacts of insect decline on ecosystem functions:** quantify the effects of observed insect declines on ecosystem functions and services, including pollination, pest control and decomposition, and the resilience of networks to species loss	Joint 3	AQ
	**Role of insects in agroecosystems:** quantify the role of insects in agroecosystems, including their role as pollinators, natural predators and decomposers, and comparing this across different farming systems, such as organic versus conventional	5	18
	**Ecosystem service values**: calculate the values of ecosystem services less well studied than pollination, including biological pest control, soil improvement, biochemical processes, and the role of key insect groups such as parasitoids, carnivorous carabid beetles and ants	Joint 6	19
	**Managing for resilient insect communities:** identify effective landscape and site‐level interventions to ensure resilience in insect communities, in managed landscapes (other than nature reserves)	Joint 6	27
Technology and Resources	**Cultivated insects:** understand the consequences of using insects as recycling agents and as food for livestock and humans, including the challenge of scaling up	1	AQ
	**Insects and climate change:** apply knowledge from entomology to inform mitigation of, and adaptation to, climate change	2	AQ
	**Insects and medicine:** develop new therapies from insects for medicinal purposes	Joint 4	28
	**Entomophagy:** evaluate the extent to which we can reduce emissions and meet protein demand by using insects as food	Joint 4	30
Pests	**Spatially integrated pest control:** integrate control strategies at both local and global scales, with involvement of all stakeholders	1	AQ
	**Invasive pests:** improve the management of non‐native and invasive species and their associated diseases	2	AQ
	**Insect pathogens:** exploit insect pathogens as alternatives to chemical pesticides for pest control	3	AQ
	**Disease vectors and climate change:** evaluate how climate change will impact vector‐borne diseases transmitted by insects, and how to mitigate these impacts	4	AQ
	**Avoiding harm to non‐target insects:** develop methods to control crop pests without harming non‐target insect species	8	8
	**Reducing pesticide exposure:** develop and expand strategies to reduce the exposure of people to pesticides, to protect human health in all countries	Joint 6	13
	**Predicting and controlling pest outbreaks:** determine drivers of pest outbreaks in agricultural, plantation and urban landscapes, and establish how they can be predicted and controlled sustainably	5	16
	**Semiochemicals and pheromones in pest management:** improve monitoring and control of pest insects using semiochemicals and pheromones	Joint 6	29
Knowledge Access	**Connecting professionals and amateurs:** stimulate and provide funds to support knowledge exchange between professional and amateur entomologists and facilitate reciprocal access to laboratory resources, literature, collections and field records	1	AQ
	**Data access:** increase the accessibility of existing entomological data, including published and unpublished work, and raw data	2	AQ
	**Identification in biodiversity hotspots:** increase the availability of insect identification guides in global biodiversity hotspots	3	11
	**Supporting entomological communities:** develop self‐supporting entomological communities in low‐income countries, particularly in entomologically diverse tropical and sub‐tropical regions	Joint 4	15
	**Phone apps:** explore the potential for phone apps to help with insect identification across a range of scenarios, including biodiversity assessments and insect monitoring	Joint 4	23
Training and Collaboration	**International capacity**: build international capacity, including identification skills, and the management of scarce funds for taxonomic research projects and training	1	AQ
	**Diversity of the entomological community** ^ϕ^: ensure that entomological research is visible and welcoming to members of ethnic minority groups and other underrepresented communities	2	AQ
	**Career pathways**: increase funding and accessibility, to enhance routes into entomology for early career researchers and those with diverse career paths.	3	7
	**Entomology in conservation**: facilitate specialist entomological support to biodiversity conservation projects on the ground, with follow‐up resources to present practical results to support conservation activities	4	26
Societal Engagement	**Online broadcasting**: make use of video content to educate and inspire about entomology, by further developing social media outlets such as the Royal Entomological Society YouTube channel, including more talks, events and contributors from around the world	1	AQ
	**School curricula**: increase the representation of insects and natural history in curricula, for science and humanities subjects	2	AQ
	**Public perceptions of insects**: encourage the public and media to engage with insects and other invertebrates in a positive way and overcome ideas about them being ‘creepy’ or ‘yucky’	3	AQ
	**Urban green spaces**: encourage urban communities to engage with local green spaces and promote their management for insect conservation	4	9
	**Government policy**: increase engagement of government policy makers with entomology and insect conservation, identify the best way to do this, and explore how entomological societies can play a more active role	5	10
	**Farming**: improve engagement with the farming community to encourage the development of practices that benefit invertebrates	6	25

*Note*: The top‐voted 10% of suggestions from Day 1 within‐theme discussions were automatically added to the final list, with their ranking from these discussions shown as a number in the ‘Day 1 results’ column, and their final status as ‘automatically qualifying’ shown as AQ in the ‘Day 2 results’ column. The next top voted 10% of suggestions from Day 1 within‐theme discussions were discussed further by all workshop participants in a Day 2 cross‐theme discussion. The ranking given to each of these in Day 1 is given within the ‘Day 1 results’ column, but they are ordered according to their final ranking during the cross‐theme discussions, shown in the ‘Day 2 results’ column. Only one priority challenge in the final list (marked ^ϕ^) passed from Stage 3 to Stage 4 as a ‘wildcard’.

The first workshop day focused on within‐theme prioritisation. In theme breakout rooms, each suggested challenge was introduced by the assigned participant, and then discussed for a maximum of 10 min, guided by a facilitator (H.E.R., S.W., L.A.N.T., S.H.L., A.W., C.D.T.) and supported by a scribe (M.P.T.G.T., S.L.B., I.B., E.S.K., M.G., N.B.Z.), who recorded key discussion points and any agreed wording changes. Following each discussion, participants (but not facilitators or scribes) independently scored the importance of the suggested challenge using their own offline spreadsheet.

At the end of Day 1, challenges in each theme were ordered by the mean rank across scorers. The top 10% of suggested challenges in each theme were automatically included in the final list of priority topic suggestions. The next highest ranking 10% in each theme went forward to the second day of the workshop, when all participants worked together in a single cross‐theme discussion. Each challenge identified for further discussion was considered in turn, guided for each theme by the same facilitator as on Day 1.

On Day 2, participants privately scored the importance of each suggested challenge following its discussion, as they had done on Day 1, and results were compiled to give an overall ranked list of suggested challenges from across all themes, to add to the final priority set. Suggested challenges discussed on Day 2 that were not ranked by any participant in their top five were removed.

The final list of challenges in entomology was therefore made up of suggested challenges that met the following criteria:

- Received higher than a specified threshold number of votes in their theme from RES members in the first round of prioritisation (Stage 3) or re‐instated following initial prioritisation by at least one participant (‘wildcards’)
- Ranked in the top 20% of suggested challenges in their theme, following discussion by participants with expertise/interest in that theme, in the second round of prioritisation (Stage 4)
- If not in the top 10% within their theme (Stage 4, Day 1), then ranked as high priority (top 5/32) by at least one workshop participant, when considered alongside suggested challenges across all 11 themes (Stage 4, Day 2)

The original suggested wording for each challenge was visible to all participants throughout the process. For publication, the steering group has edited the text of the final set of priority challenges, for consistency of formatting and clarity of understanding.

### Data analysis and visualisation

At each stage of the process (Stage 1 survey, Stage 3 survey, and Stage 4 workshop participation), we compared the distributions of participant age (7 categories), gender (male/female) and country composition (for the 10 countries that have >10 RES members) with the RES membership (excluding journal editorial teams and special interest group members), using chi‐squared tests. The RES did not have data on entomological role, years active in entomology, or journal preferences, and so the responses to these within Stage 1, Stage 3 and Stage 4 are presented without comparison.

We assessed the relationship between the number of times a challenge was suggested in Stage 1, prior to amalgamating duplicates in Stage 2, and its likelihood of reaching the final list of priorities, using a generalised linear model with presence/absence in the final priority list as the response variable, and number of original suggestions as the predictor variable, using the family ‘binomial’ and a ‘logit’ link.

We used R version 4.1.2 (R Core Team, 2021), R Studio version 2021.9.1.372 (R Studio Team, 2021), and the packages ‘ggplot2’ (Wickham, 2016), ‘dplyr’ (Wickham et al., 2021), ‘tidyr’ (Wickham & Girlich, 2022), ‘tibble’ (Müller & Wickham, 2021), ‘DHARMa’ (Hartig, 2022), and ‘gridExtra’ (Auguie, 2017) to organise, plot, and analyse the data.

## RESULTS

Key results are outlined here, with additional details included within Appendix S3.

### Involvement and scope

#### Stage 1: Gathering suggested challenges

A total of 189 RES members (11.8% of the total RES membership at the time) completed the initial online survey (Stage 1), contributing 710 topic suggestions (Figure 1). Respondents included representatives from 24 countries (of which 11 countries had two or more respondents) and ranged in age from 18–24 to 75+ (Figure S2). A majority of respondents were male (143), and UK based (141), and reflected the 2020–2021 RES membership profile in age, gender and where they live in the world (*p* > 0.4 in all comparisons) (Appendix S3; Figure S2). Respondents varied from 0–10 to 50+ years of activity within entomology, with a reasonably even spread of responses across all time periods (Figure S3).

#### Stage 3: Prioritising suggested challenges

After processing and amalgamation of duplicate ideas (Stage 2), 472 suggestions were put forward for the first stage of prioritisation (Figure 1). One hundred and eighteen members (9.4% of those who received the survey) completed Stage 3. Responses were received from members resident in 15 different countries (of which eight had two or more respondents), across all age ranges, and were representative of the full RES membership (*p* > 0.3 in all comparisons) (Appendix S3). Respondents in the second survey tended to have had fewer years of activity within entomology (earlier career), on average, than those who replied to the first survey, with 0–10 years being the most common period of involvement, and a slightly less male‐biased gender balance (68% male) (Figure S3).

#### Stage 4: Prioritising shortlisted challenges

Online voting led to 235 suggestions being put forward to Stage 4, and seven wildcards were reintroduced on request from participants (Table S2), giving 242 suggestions in total. The workshop involved 54 participants (including RES members, workshop organisers, scribes, and facilitators), of whom 37 were entitled to vote (see Table S1). The 37 voting participants represented a wide cross‐section of the membership (Figures S2 and S3) and was overall younger and more female skewed than the RES membership as a whole (age: χ^2^ = 31.70, df = 6, *p* < 0.001; gender: χ^2^ = 13.52, df = 1, *p* < 0.001), with the majority of participants under the age of 54, and an approximately equal male/female split. In line with the RES membership as a whole, the majority of participants were UK based (χ^2^ = 5.0898, df = 10, *p* = 0.89), although there were representatives from seven different countries.

Participants in each of the above stages were most frequently University‐affiliated academics and more likely to choose ‘Ecological Entomology’, ‘Insect Conservation and Diversity’, and ‘Agricultural and Forest Entomology’ as their preferred RES journals, although the full range of roles and journal preferences were always represented (Figure S3).

Day 1 within‐theme discussions contributed 31 suggestions to the final list, including one that made it to Stage 4 as a ‘wildcard’ (‘Day 1 results’ in Table 1); the across‐theme discussion on Day 2 added an additional 30 suggestions (‘Day 2 results’ in Table 1). The final list of RES Grand Challenges in Entomology included 61 challenges.

### Emerging themes and priority challenges

**FIGURE 2:**
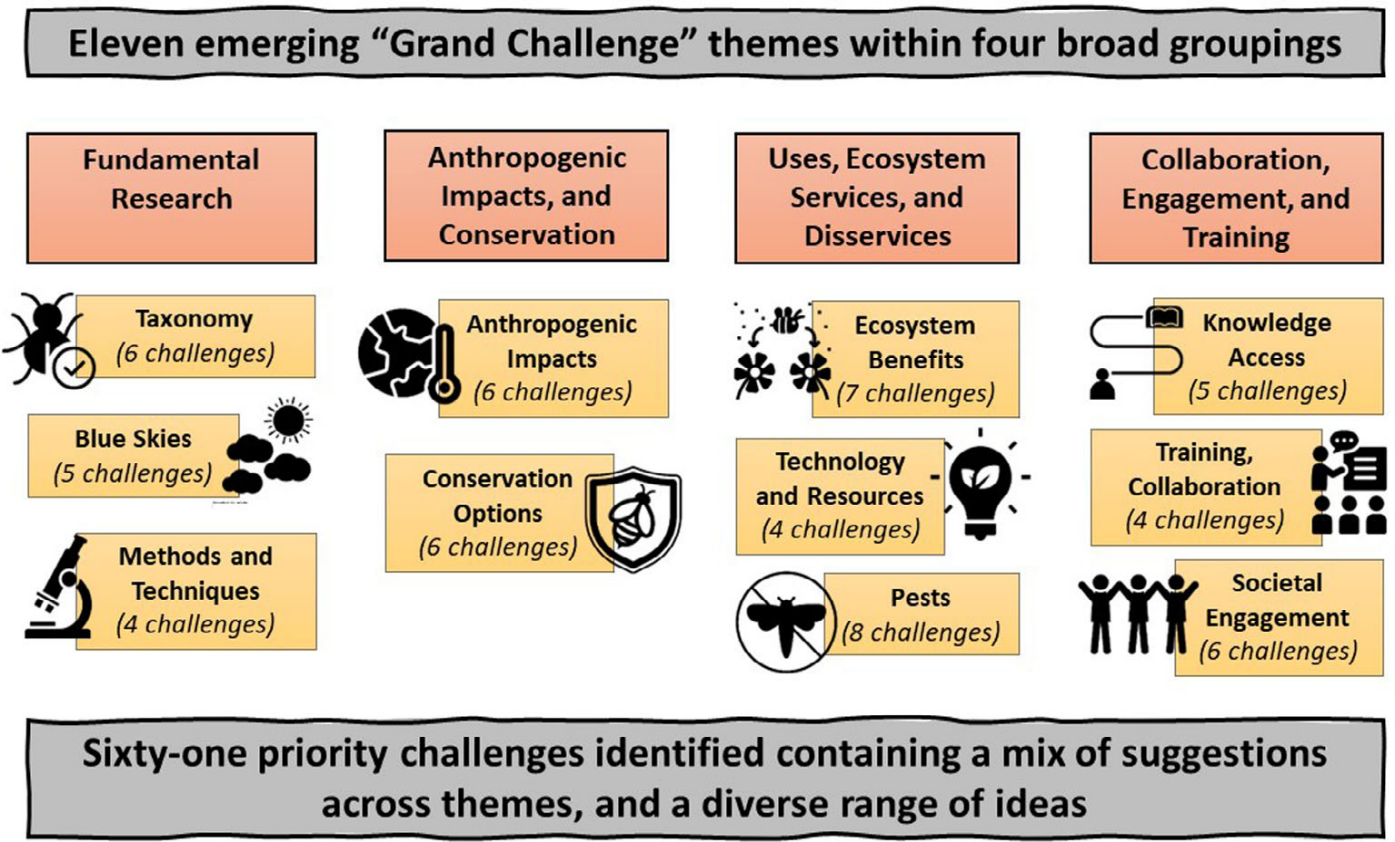
Schematic illustration of 11 “Grand Challenge” themes that emerged from the prioritisation process (light orange boxes, with grey outlines), organised within four broad topic groupings (dark orange boxes, with black outlines). The final list of priorities included 61 challenges spread across these themes. Number of challenges within each theme are shown in parentheses under each theme heading. All images are from NounProject.com. See Supplementary Materials Appendix S3 for a full list of credits.

Eleven broad “Grand Challenge” themes emerged, which can be organised into four groupings of related themes (Figure 2, Table S2). These are defined as:

- Fundamental research
  - Taxonomy: Taxonomic research and understanding of what insect diversity exists
  - Blue skies: Fundamental science research ideas, without an immediate practical application
  - Methods and techniques: Developing research techniques and methods to facilitate entomological research
- Anthropogenic impacts and conservation
  - Anthropogenic impacts: Changes in insect communities, causes of changes
  - Conservation options: Possible conservation strategies
- Uses, ecosystem services, and disservices
  - Ecosystem benefits: Benefits we get from insects within ecosystems
  - Technology and resources: Insects as inspiration for technology, and as a material/resource
  - Pests: Insects as pests: problems and solutions
- Collaboration, engagement, and training
  - Knowledge access: Access to research resources and knowledge
  - Training and collaboration: Career development, training, and sharing of ideas, for entomologists
  - Societal engagement: Engagement of wider society

The final list of 61 priority challenges contained a mix of suggestions across themes, and a diverse range of ideas (Figure 2, Table 1). There was a positive relationship between the number of survey respondents who initially suggested a challenge and the likelihood of it making the final priority list (*z* value = 2.722, *p* = 0.00648; Appendix S3, Figure S4).

#### Fundamental research

Priority challenges in this group ranged across several fundamental science topics, with a strong emphasis on increased funding and capacity for such topics. There was also a focus on harnessing new technologies to better monitor insects and to extend networks of monitoring sites (Figure 2, Table 1, Table S2). Emerging topics included ecological networks, insect pollinator and plant interactions, insect life‐history research, and the role of insects in ecological functions. The need to fund curiosity‐driven ‘blue skies’ research and taxonomy—particularly in geographical regions with large proportions of undescribed fauna—were prioritised. This call for taxonomy funding was coupled with a desire to develop ways to encourage people to become taxonomists and to support those wishing to embark on a career in taxonomy, particularly in an era when collecting is becoming more difficult due to legal and ethical challenges. Suggestions relating to new technologies emerged clearly, including increasing digitisation of museum collections, developing automated identification techniques to allow insect identification by non‐experts, promoting the integration of molecular and classical taxonomy, and the use of genetic approaches to inform our understanding of the impacts of environmental change on insects. Development of insect biodiversity monitoring techniques and establishment of a global network of insect monitoring sites to allow long‐term monitoring were also prioritised.

#### Anthropogenic impacts and conservation

Priorities included a strong focus on quantifying, understanding, and reversing insect declines and community changes, and a range of landscape‐scale approaches to help address this in different contexts and habitats (Figure 2, Table 1, Table S2). Specific topics that were highlighted included the need to find out whether insect declines are happening globally, to understand what the main drivers of insect population changes are and whether these vary across biomes, and to determine insects' resilience to impacts and whether there are tipping points. There was also a focus on understanding the impact of climate change on insect movement and interactions, and the ecological consequences of any loss or changes in insect diversity as a result of anthropogenic impacts. Prioritised options for conservation included considering insects in landscape scale conservation projects and improving the design of agricultural landscapes and urban areas—including options for ‘retro‐fitting’ urban areas—to make them more insect‐friendly. Developing understanding of the value of habitat corridors for insect movement and persistence, and the impacts of rewilding projects for insects were also highlighted.

#### Uses, ecosystem services, and disservices

Key emerging topics included a strong desire to better understand the role of insects in ecosystems, to develop their use to provide services for people, and also to find ways to increase peoples' awareness of the role of insects. Developing a better understanding of the role of insects as pests, and the need to find more sustainable ways of monitoring and controlling pest outbreaks were also prioritised (Figure 2, Table 1, Table S2). There was a call for greater consideration of soil insect biodiversity and its role in promoting soil health, the role of insects in agroecosystems more broadly and the value of non‐pollination services in particular, and also to determine roles of understudied taxa in ecosystem functioning. The need to understand how ecosystem services could change as a result of insect declines, and the need to find ways to promote resilience in ecosystem service provisioning were also highlighted. The potential contribution of insects to recycling, as food for humans and livestock, part of new medical therapies, and as a strategy in battling climate change, were also deemed top priorities.

Pest‐related priorities focused on trying to better understand the impacts of climate change on disease and the drivers of pest outbreaks in different landscapes. They also included options for improving pest control through the use of insect pathogens, semiochemicals, pheromones, and other more environmentally friendly approaches to insect control. In addition, the challenges highlighted a need for better integration of pest control approaches across spatial scales and including all stakeholders.

#### Collaboration, engagement, and training

A wide variety of suggestions for increasing entomological awareness, appreciation, and skills across a broad range of sectors of society were prioritised. These ranged from school children to government, including professional scientists, farmers, amateur entomologists, and the general public, both in the United Kingdom and globally (Figure 2, Table 1, Table S2). Ideas that emerged strongly related to increased public appreciation of insects, including inclusion of insects and natural history in school curricula, developing campaigns to overcome ideas about insects being ‘creepy’ or ‘yucky’, and encouraging local communities in urban areas to educate people regarding insect‐friendly management practices in local green spaces. The need to engage government, policy makers, and farmers about insect conservation was also prioritised. There was a strong desire to help support people to access entomological training, particularly for those from underrepresented or disadvantaged groups, including early career researchers, those from diverse career paths, minority ethnic groups, and international researchers. The need to build international capacity, to increase the availability of identification guides and open access publication (with provision to ensure affordability for entomologists from all countries), to communicate between professional and amateur entomologists, and to support long‐term self‐sustaining projects and entomologist communities around the world, were all prioritised. One of the most highly voted suggestions within this grouped theme was a request for the RES itself to increase the use of its YouTube channel, to help support entomological education, and to inspire new research directions and collaborations.

## DISCUSSION

### Emerging priorities

Research‐focused challenge areas included enhancing understanding of insect diversity, form and function (including biodiversity, communities, networks, interactions, pests, and taxonomy); anthropogenic impacts on insects (including declines, losses, agriculture and urban impacts, and climate change); and developing conservation solutions (including rewilding and landscape management). Priorities related to engaging wider society with insects and informing them of the key role insects play in human well‐being also emerged strongly from the exercise. Some of these issues—such as pests—have been relatively well studied throughout the 20th century. However, others— such as anthropogenic impacts and conservation solutions—have been considered increasingly in recent years, and some are only just emerging on the entomological agenda. This includes issues that have recently hit the headlines, such as ‘insect declines’ (Didham et al., 2020; van Klink et al., 2020), entomophagy (de Carvalho et al., 2020), and the lack of natural history in education (Tewksbury et al., 2014). In the United Kingdom, a new ‘Natural History’ qualification for 16‐year‐olds has recently been announced, which provides one opportunity to enhance entomological education, but there are many others, throughout educational stages from pre‐school onwards.

A Grand Challenges Agenda for entomology recently conducted by the ESA identified three main challenge areas: (1) vector‐borne diseases and their impacts on human health; (2) invasive insect species, including global trade, biodiversity, climate change; and (3) sustainable agriculture, including addressing global hunger, food security, and natural resource preservation (https://entomologychallenges.org/). While there are several parallels in the foci chosen by the RES and the ESA exercises—including the role of insects in agriculture, food security, and consideration of biodiversity and climate change—the RES exercise resulted in a broader and more diverse set of challenges. Vector‐borne disease, invasive species, and global trade are much less prominent in the set of challenges identified by RES members, whereas topics related to needs for monitoring, training, encouragement, and funding to enable entomology to achieve its potential, in contributing to societal goals, were much more strongly highlighted. There was also consideration of the need to address diversity issues in entomology and to increase access to knowledge and training for disadvantaged groups, as well as giving greater consideration to supporting equitable interactions between scientists around the world. Among many possible reasons for this, the differences in scope could perhaps have been affected by setting differing aims for the end result (in terms of number of suggestions generated or focused on, and the specificity of these), the greater number and diversity of participants involved in the RES process, or a difference in priorities between the two societies, perhaps influenced by their geographic focus. Owing to the differences in approach taken, the ESA and RES lists of priorities are highly complementary, and together offer a diverse range of options for how to direct future actions.

### Shortcomings and possible biases

Conducting a prioritisation process such as the RES Grand Challenges exercise has the advantage of being able to gather thoughts and opinions from a wide range of people with varying expertise. However, the contents of the final list inevitably depend on the views of participants at each stage, and so are vulnerable to the effects of selection and participation bias.

Biases could be apparent at various stages from the initial population who were invited to participate, the set of people who chose to complete the online surveys, and who chose to attend and speak out in the on‐line meetings. The RES is a UK‐based organisation with a fee for membership. Although it has members from over 50 countries, its membership is dominated by UK‐ and European‐based entomologists, with few members from tropical and Global South countries; the majority is male (76%) and over 45 years old (73%) (based on 2020 membership figures). Without access to a global census of entomologists, we cannot be sure to what extent this represents the wider entomological community and their views, but it is likely that some topics—for example, those related to tropical systems—could have been under‐represented because of biases in the initial selection of invited participants. However, our analysis showed that respondents contributing to Stages 1 and 3 were representative of the current RES membership. Although our survey response rates at Stages 1 and 3 were relatively low (11.8% and 9.4%, respectively), this is expected from online surveys and falls comfortably within the range reported from a meta‐analysis of published survey response rates by Shih and Fan (2008).

The on‐line workshop involved approximately even numbers of participants identifying as male or female, with a skew towards younger age groups, perhaps as a result of availability to participate and also the reward of co‐authorship potentially encouraging high engagement from early career academics. Although not fully representative of the current RES membership, this was arguably a more representative mix of voices from across the entomological community as a whole and captures the direction of change in the RES membership towards greater diversity and inclusion.

In addition to a skew in socioeconomic traits of respondents, there was also a skew towards responses from members who chose ‘Insect Conservation and Diversity’, ‘Ecological Entomology’ and ‘Agricultural and Forest Entomology’ journals as their most read, which could have substantially influenced the final list of priorities. The RES does not collect data on the topic interests and expertise of its members, and so it is difficult to know whether this bias represents the current membership of the society, or a bias in who we recruited for the project. However, information showing monthly downloads from the seven RES journals from January 2017 to May 2022 (available from Wiley Online Library) shows that ‘Ecological Entomology’ was consistently the journal experiencing the most downloads. Ecology, conservation, and landscape‐scale ideas came through strongly in the final list of priority topics, and so it should be acknowledged that this was potentially influenced by our recruitment profile.

The process was conducted entirely online owing to the COVID‐19 pandemic, rather than including an in‐person workshop as had been planned, and as is common in similar exercises (Sutherland et al., 2011). Recent research in experimental psychology demonstrates that although groups using video‐conferencing are not able to produce new creative ideas as easily, they are at least as effective as in‐person groups when it comes to selecting which ideas to pursue (Brucks & Levav, 2022). Also, online workshops can help to enhance accessibility and increase inclusivity (e.g. similar to the benefits recorded for online conferences; Sarabipour, 2020).

Topics suggested by multiple contributors at Stage 1, which were amalgamated in Stage 2 before voting in Stages 3 and 4, were more likely to be chosen for the final list of priorities than those suggested by fewer people. This suggests that despite lower numbers of participants in later stages of the process, and potential skew in the demographics of these groupings, the choices of later stage participants reflected the ideas that came through strongly across the wider membership at the first stage of the process.

### Where next?

The emergence of such a wide range of priority topics, across 11 very different themes, reflects the breadth of entomology as a discipline. This invites consideration of what entomologists can and should achieve in the coming decades, as well as the role that entomological societies—including the RES specifically—can play in this.

Similar collaborative exercises to identify knowledge needs or challenges have provided agendas to shape future research programmes (Dicks, Bardgett, et al., 2013 on sustainable agriculture and Loury et al., 2021 on the management of migratory fish species in Cambodia) or helped to shape responses to an emerging or urgent problem (Morris et al., 2017 on bark beetle outbreaks). Jucker et al. (2018) found that for 45 of the 100 questions on global biodiversity conservation prioritised by Sutherland et al. (2009), >100 review papers had subsequently been published related to each, demonstrating significant research effort, potentially catalysed by the process. For some previous collaborative prioritisation exercises, as for the entomology challenges reported here, items in the final list have gone beyond research questions, to encompass specific engagement activities or policy priorities. For example, the priority knowledge needs for wild insect pollinator conservation compiled by Dicks, Abrahams, et al. (2013) included ‘Training for conservationists, agronomists and land managers on pollinator ecology and conservation’ as a high priority, and ‘New agri‐environment options that provide nesting resources for bees’, a policy priority that has not yet been achieved, to our knowledge. In the present study, we deliberately kept the focus broad by asking for topics on which ‘entomologists should focus their efforts’, rather than asking for answerable questions or knowledge needs. The result is a particularly diverse list, which we think has a range of uses.

Many of the challenges identified by this process can be acted upon by entomological researchers. Several lend themselves to detailed scientific reviews, which would be timely. For example, the top challenge in the ‘Ecosystem benefits’ theme is ‘Insects’ contributions to people’, which is equivalent to ecosystem services provided by insects, but using the language of the Intergovernmental Science Policy Platform on Biodiversity and Ecosystem Services (Díaz et al., 2018). We do not know of a comprehensive overview of this in the literature for all insect groups, although several recent papers have focused on specific taxa or contexts (Brock et al., 2021, aculeate wasps specifically; Elizalde et al., 2020, social insects more widely; Macadam & Stockan, 2015, freshwater insects; and Saunders, 2018 within agricultural systems), while Noriega et al. (2018) discuss trends in research on ecosystem services provided by insects. Other priority challenges demand research and methods development, rather than literature review. The same theme identifies ‘Ecosystem service values’, particularly beyond the value of pollination, as a priority challenge. To our knowledge, Losey and Vaughan (2006) were the last authors to attempt a valuation of services provided by all insects and produced an estimated value of $57 billion per year for the United States of America alone, even though this was based on a limited number of ecosystem services. Valuation methods and relevant datasets have developed a lot since then. For example, Beynon et al. (2015) estimated the value of dung beetles to the UK cattle industry at £367 million per year. It would be immensely useful to repeat Losey and Vaughan's exercise for all insects, particularly in the context of the value of losses to insects (e.g. >$470 billion per year, estimated by Culliney, 2014).

Many of our priority challenges demand action by the wider community of amateur and professional entomologists, or learned societies themselves, such as the RES. Following this exercise, the RES has already begun to align its annual ‘Ento’ scientific conference programmes with the identified Grand Challenge themes and will continue to do so for the foreseeable future. In addition, RES Special Interest Groups (SIGs) have all been asked to consider how they can best align their work with the workshop findings, and a new Policy SIG has been organised to address how Grand Challenges can be used to engage with policymakers working in entomological areas. In direct response to a call for improved engagement via YouTube, the RES has already increased the level of content on its own channel (https://www.youtube.com/channel/UC7zqYiJ5Y1nkqcydJXuRvmQ), including uploading more recorded scientific talks. The Society continues to update its engagement with and monitoring of equality, diversity and inclusion (EDI) initiatives and impacts of its work. Results of the Grand Challenges prioritisation exercise have been shared with the ESA and are being shared with the wider entomological community through additional publications. For example, following the outline of methods and results provided within this current paper, there are plans to discuss aspects of the priority list in more detail, and consider topic‐specific next steps in follow‐up editorial articles in RES journals.

The RES ‘Grand Challenges in Entomology’ initiative aimed to inform the direction of entomology, consider what entomologists can do for society and develop an agenda of topics for the 21st century. Over 200 entomologists collaborated in a multi‐stage process of developing, discussing, and prioritising over 700 challenges. We encourage readers to consider the list of challenges as a call to action, whatever their role in the future of entomology. If all these challenges can be addressed, the science of entomology will have a diverse, vibrant and influential future, and remain an important discipline in the natural and environmental sciences, and in natural history in the decades ahead. If entomology can successfully deliver broad research, policy action, and changes in societal attitudes, it can provide a thriving future for insects, safeguarding vital resources, ecosystem services, and biodiversity throughout the 21 century.

## AUTHOR CONTRIBUTIONS

**Sarah H. Luke:** Conceptualization (equal); data curation (lead); formal analysis (lead); investigation (equal); methodology (equal); project administration (lead); visualization (lead); writing – original draft (lead); writing – review and editing (lead). **Helen Elizabeth Roy:** Conceptualization (supporting); funding acquisition (equal); investigation (supporting); methodology (supporting); project administration (supporting); resources (supporting); writing – original draft (supporting); writing – review and editing (supporting). **Chris D. Thomas:** Conceptualization (supporting); funding acquisition (equal); investigation (supporting); methodology (supporting); project administration (supporting); resources (supporting); writing – original draft (supporting); writing – review and editing (supporting). **Luke Tilley:** Conceptualization (supporting); funding acquisition (equal); investigation (supporting); methodology (supporting); project administration (supporting); resources (supporting); writing – original draft (supporting); writing – review and editing (supporting). **Simon Ward:** Conceptualization (supporting); funding acquisition (equal); investigation (supporting); methodology (supporting); project administration (supporting); writing – review and editing (supporting). **Allan Watt:** Conceptualization (supporting); funding acquisition (equal); investigation (supporting); methodology (supporting); project administration (supporting); resources (supporting); writing – original draft (supporting); writing – review and editing (supporting). **Manuela Carnaghi:** Data curation (supporting); formal analysis (supporting); investigation (supporting); methodology (supporting); project administration (supporting); writing – review and editing (supporting). **Coline C. Jaworski:** Data curation (supporting); formal analysis (supporting); investigation (supporting); methodology (supporting); project administration (supporting); writing – review and editing (supporting). **Maximillian P.T.G. Tercel:** Data curation (supporting); formal analysis (supporting); investigation (supporting); methodology (supporting); project administration (supporting); writing – review and editing (supporting). **Charlie Woodrow:** Data curation (supporting); formal analysis (supporting); investigation (supporting); methodology (supporting); project administration (supporting); writing – review and editing (supporting). **Susmita Aown:** Investigation (supporting); writing – review and editing (supporting). **Jennifer Alessia Banfield‐Zanin:** Investigation (supporting); writing – review and editing (supporting). **Sarah L. Barnsley:** Project administration (supporting); writing – review and editing (supporting). **Iris Berger:** Project administration (supporting); writing – review and editing (supporting). **Mark J. F. Brown:** Investigation (supporting); writing – review and editing (supporting). **James C. Bull:** Investigation (supporting); writing – review and editing (supporting). **Heather Campbell:** Investigation (supporting); writing – review and editing (supporting). **Ruth A.B. Carter:** Investigation (supporting); writing – review and editing (supporting). **Magda Charalambous:** Investigation (supporting); writing – review and editing (supporting). **Lorna Jane Cole:** Investigation (supporting); writing – review and editing (supporting). **Martin Ebejer:** Investigation (supporting); writing – review and editing (supporting). **Rachel A. Farrow:** Investigation (supporting); writing – review and editing (supporting). **Rajendra S. Fartyal:** Investigation (supporting); writing – review and editing (supporting). **Miriam Grace:** Project administration (supporting); writing – review and editing (supporting). **Fiona Highet:** Investigation (supporting); writing – review and editing (supporting). **Jane K Hill:** Investigation (supporting); writing – review and editing (supporting). **Amelia S.C. Hood:** Investigation (supporting); writing – review and editing (supporting). **Eleanor S. Kent:** Project administration (supporting); writing – review and editing (supporting). **Frank Krell:** Investigation (supporting); writing – review and editing (supporting). **Simon Leather:** Investigation (supporting); writing – review and editing (supporting). **Daniel J. Leybourne:** Investigation (supporting); writing – review and editing (supporting). **Nick Littlewood:** Investigation (supporting); writing – review and editing (supporting). **Ashley Lyons:** Investigation (supporting); writing – review and editing (supporting). **Graham Matthews:** Investigation (supporting); writing – review and editing (supporting). **Louise Mc Namara:** Investigation (supporting); writing – review and editing (supporting). **Rosa Menéndez:** Investigation (supporting); writing – review and editing (supporting). **Peter Merrett:** Investigation (supporting); writing – review and editing (supporting). **Sajidha Mohammed:** Investigation (supporting); writing – review and editing (supporting). **Archie Murchie:** Investigation (supporting); writing – review and editing (supporting). **Michael Noble:** Investigation (supporting); writing – review and editing (supporting). **Maria‐Rosa Paiva:** Investigation (supporting); writing – review and editing (supporting). **Michael J. Pannell:** Investigation (supporting); writing – review and editing (supporting). **Chooi‐Khim Phon:** Investigation (supporting); writing – review and editing (supporting). **Gordon Port:** Investigation (supporting); writing – review and editing (supporting). **Charlotte Powell:** Investigation (supporting); writing – review and editing (supporting). **Stewart Rosell:** Investigation (supporting); writing – review and editing (supporting). **Francisca Sconce:** Project administration (supporting); writing – review and editing (supporting). **Chris Shortall:** Investigation (supporting); writing – review and editing (supporting). **Eleanor M Slade:** Investigation (supporting); writing – review and editing (supporting). **Jamie P. Sutherland:** Investigation (supporting); writing – review and editing (supporting). **Jamie C. Weir:** Investigation (supporting); writing – review and editing (supporting). **Christopher David Williams:** Investigation (supporting); writing – review and editing (supporting). **Natalia B. Zielonka:** Project administration (supporting); writing – review and editing (supporting). **Lynn Vanessa Dicks:** Conceptualization (equal); data curation (supporting); formal analysis (supporting); funding acquisition (equal); investigation (equal); methodology (equal); project administration (supporting); resources (supporting); supervision (lead); visualization (supporting); writing – original draft (supporting); writing – review and editing (supporting).

## CONFLICT OF INTEREST STATEMENT

Luke A. N. Tilley, Sajidha Mohammed, and Francisca Sconce are employed by the Royal Entomological Society (RES); Helen E. Roy, Chris D. Thomas, Simon Ward, Allan Watt, Lynn V. Dicks and Jane K. Hill play senior roles in the management of the RES, including as trustees, president, or former president; and the majority of co‐authors are members of the RES.

## Supporting information


**Data S1:** Supporting Information

## Data Availability

The data that supports the findings of this study are available within the article. Further details are available from the corresponding author upon reasonable request.
