# Supplementary material for: Grand challenges in entomology: Priorities for action in the coming decades
Source: Insect Conserv Divers. 2023 Mar 20;16(2):173–89. doi: 10.1111/icad.12637 (PMC10947029; doi:10.1111/icad.12637)
Supplement: Supplementary file 1 — Data S1: Supporting Information [file ICAD-16-173-s001.docx]

**Supplementary Materials for Luke *et al.* (2023) Grand challenges in entomology: Priorities for action in the coming decades**

**Appendix 1 –** Additional methods details

The collaborative prioritisation exercise was designed and implemented by a steering group, made up of the incumbent/incoming presidents of the RES at the time (CDT, HER), members of the RES Council (LANT, SW, AW), and researchers from the University of Cambridge (LVD, SHL) (see Table S1).

***Stage 1 – Gathering suggested challenges***

- To help guide members when suggesting challenges, we gave some examples of suggestions that would be considered too broad, too specific, or ‘just right’ (e.g., Too broad: ‘What is driving insect decline and how do we stop it? Too specific: Is a change in temperature in the New Forest leading to the loss of species X? Just right: How important is climate change as a driver of insect decline on tropical mountains?).
- The Qualtrics survey used to collect challenge suggestions and answers to demographic questions was accessed by respondents using a link sent by email. Invitations to participate were also sent by post to individuals known to the RES who were unable to, or preferred not to, receive emails.

***Stage 2 - Processing suggested challenges***

- Developing a thematic framework for the suggested challenges involved four members of the research team independently reading the list of topic suggestions, considering the key themes that they covered, and thinking about how best to organise the list into themed groupings. Through comparison of the independent frameworks and subsequent discussion, a final thematic framework was agreed between the four members of the team.
- Agreement in how the challenges were sorted across the thematic framework was assessed using Kappa analysis. This was checked after each successive subset of 50 challenges was sorted, using Fleiss’ Kappa for multiple raters (Fleiss et al., 2004) calculated using the R package “irr” (Gamer et al., 2019). To assess whether agreement across raters was different from chance agreement, we checked p-values for the overall rating and for the individual theme groupings (p<0.05 indicating the result is unlikely to have occurred by chance), and the overall Kappa value (between 0.4 and 0.75 indicates fair to good inter-rater agreement beyond chance, and 0.75 or higher indicates excellent agreement, Fleiss et al., 2004). When these target values were not met, discrepancies were discussed, and consensus reached about the criteria for thematic assignment. P-values of <0.001 were achieved overall and for all separate values, with an overall Kappa value of 0.67 after sorting 100 suggestions. After this point, a single team member (SHL) sorted the remaining suggestions into the agreed themes.
- Once suggested challenges had been sorted into themes, a single member of the research team (SHL) amalgamated duplicate suggestions. In cases where the same idea was conveyed using very similar wording, only one version was retained. In some cases, wording of the retained suggested challenge was adjusted to capture aspects of highly related ideas. Although this process was completed by a single researcher, a full record of all amalgamations was made available to all participants at Stage 3 and Stage 4 of the process, so all participants in the prioritisation stages had the opportunity to check and query amalgamation decisions.
- The final processing step of moving some of the suggestions between themes helped to balance the time available for consideration of each suggested challenge during Stages 3 and 4. Only suggested challenges with some ambiguity about which theme was most appropriate were reallocated to another theme.

***Stage 3 – Prioritising suggested challenges***

- When completing the Qualtrics survey for prioritising suggested challenged, participants first selected a theme they were interested in, and/or felt they had expertise in, and then were randomly assigned to a second theme. This design allowed respondents to use their own particular expertise, whilst also ensuring that all themes were reviewed by an approximately equal minimum number of people, and by a mixture of experts and other entomologists (ensuring breadth in the prioritisation process).
- Within each of the themes they looked at, participants were asked to select the highest priority 10%. The number to be selected (rounded to the nearest whole number) was predefined as a validation step in the survey so participants could not progress without selecting the correct number. For example, participants were required to select four from a theme that included 38 suggestions, or five from a theme with 53 suggestions. In each case, it was made clear to participants how many to choose, and they could only progress once the correct number had been chosen. Priority was defined as “*Priority topics on which you think entomologists should focus their efforts over the coming years and decades*”, and *“topics around which a programme of activities or research could be designed”* (as in Stage 1).
- In cases where suggested challenges were an amalgamation of several original ideas (see Stage 2), the newly worded/combined challenge was listed, and a link provided to the originally proposed challenges. Participants had the opportunity to request clarification, suggest re-wording, or add comments to any of the suggested challenges.

***Stage 4 – Prioritising shortlisted challenges***

- The second round of prioritisation was conducted by respondents from Stage 3 who volunteered, and were available, to attend a two-day online workshop (see Supplementary Materials Figure S1 for a summary of the process). They represented a self-selecting group of entomologists with a strong interest in the process (see Supplementary Materials Table S1). From their responses to demographic questions, the steering group judged that they comprised a diverse range of respondents, and there was no need to recruit additional participants to increase representation of any particular demographic groups (see Results).
- Workshop participants were allocated to between one and three themes, aligned as closely as possible with their interests. All participants were allocated to their ‘chosen theme’ in Stage 3, or the next most closely related theme where numbers were unbalanced across theme groups.
- The collated data from Stage 3 shared with participants ahead of the Zoom workshop comprised a full list of suggested challenges considered in Stage 3, organised by theme, and presented in descending order according to the number of Stage 3 votes received. A clear cut-off was set for the number of votes below which suggested challenges would not be taken further in the process (Supplementary Materials Table S2).
- For most themes, challenges voted for by at least two people in Stage 3, or if >30 voters within a theme, by the closest threshold to 10% of voters, were proposed for discussion at the workshop. For themes with a small number of voters (<15) from Stage 3, a threshold number of votes for workshop discussion was set at either 1 or 2, so that at least 40% of the originally proposed challenges went through, except in one case (‘Blue skies’). For ‘Blue skies’, a threshold of two votes was used, putting 33% of the suggestions through to Stage 4 (i.e., <40%), because many suggestions had a single vote; a threshold of 1 vote (i.e., at least 40%) would have led to 36 suggestions being considered at Stage 4 (60% of those from Stage 3), making this the biggest theme, based on the individual opinions of 13 voters.
- We accepted no more than an average of one recalled ‘wildcard’ suggestion per participant.
- The first day of the workshop involved within-theme prioritisation. After a general introduction to the process, participants were split into five or six parallel breakout rooms according to their allocated themes. The majority of themes were discussed in a single 2.5-hour session, with breaks.
- All voting participants (see Table S1) were asked to introduce some of the suggestions during the workshop, to ensure a diversity of voices. Suggestions to introduce were assigned at random, but never to the original suggester. Participants were asked to read their allocated suggestions and prepare to say a few words about each one, and how they felt about it as a challenge in entomology, to open discussion.
- One theme, ‘Pests’, had the longest list of suggested challenges (34; see Table S2) and ran throughout the day to enable sufficient discussion time, so its participants only discussed a single theme. Most participants discussed two themes, during separate sessions in the morning and afternoon. The ‘Knowledge access’ and ‘Technology and Resources’ themes had the shortest lists (see Table S2) and were combined into a single discussion session, whose participants therefore discussed three themes together. Theme allocations were designed so the afternoon discussion groups were not composed of exactly the same people as the morning discussions.
- Each suggestion was discussed in turn. Following discussion, the importance of the suggestion was scored privately by each participant, with a unique score between 0 (lowest priority) and 100 (highest priority). Scoring was conducted independently and anonymously, using individual scoresheets in Excel, distributed before the workshop.
- The facilitator and scribe did not score, their primary roles being to enable all voices to be heard, to encourage each challenge to be given a similar length of time for discussion, and to help with correction of any factual inaccuracies.
- At the end of Day 1, each participant’s individual scores were ranked (to ensure that the views of different participants were weighted equally), and the suggested challenges in each theme were ordered by mean rank across scorers, to give an overall ranked list of suggested challenges within each theme.
- During the Day 2 discussions, all participants could see the suggestions that were automatically selected from Day 1 (AQ, Table 1), and people were encouraged to consider and discuss cross-over or duplication among themes.
- After all the challenges that had been put through from Day 1 for further discussion had been considered, participants’ individual scores were ranked, and the suggested challenges ordered by mean rank across scorers, to give an overall ranked list of suggested challenges from across all themes, to add to the final priority set. As there was no obvious cut-off point in the mean ranks, we included in the final list all challenges that ranked in the top five for any individual participant (5/32).

***Data analysis and visualisation***

- In each analysis, demographic data were unlinked from individual identities and suggested challenges or challenge-related responses, and total counts within categories were considered.

**Supplementary Materials Appendix 2** - The first Qualtrics survey, which was open to RES members between 29th October and 20th November 2020.

***First page***

**The Royal Entomological Society Grand Challenges Project aims to identify priority topics for entomological research and activities over the coming years and decades.**

We will work with you to distil a set of priorities, and use them to guide the agenda of the Royal Entomology Society and inform the activities of our members.

Priority topics will be proposed and selected by members of the Royal Entomological Society (RES), RES journal editors and editorial board members, and RES Special Interest Group associates, through a multi-stage consultation process. This process is led by researchers at the University of Cambridge, in collaboration with the RES Council.

At this first stage, we are gathering your suggestions for priority topics. Once you’ve thought of your ideas, it should take no more than 10 minutes of your time to complete the survey. If you have ideas, and wish to take part, please click ‘next’ to read more about the project and give your consent.

***Second Page***

**Participant Information**

Before you decide to submit ideas, please read the following information. Contact Dr Sarah Luke (shl47@cam.ac.uk) at the University of Cambridge if anything is unclear, or you want to know more.

**What is the research about?** - The Royal Entomological Society is interested in distilling a set of priorities that it will use to guide its agenda and activities over the next several years. To do this, it would like to gather the views of its members, journal editors and editorial board members, and Special Interest Group associates, and involve them in a prioritisation process to select a final list of “Grand Challenge” priority topics. The prioritisation process will be led by researchers at the University of Cambridge, in collaboration with members of the RES Council and will follow established research methods. This questionnaire forms the first stage of the process where members are invited to submit their ideas for priority topics.

**Why have I been asked to participate?** – You are listed within the RES records as a member, journal Editor/Associate Editor or Special Interest Group Associate, and so we would very much like to hear your suggestions. We hope to collect as a wide a range of views as possible from these groups.

**What does the survey entail?** – After giving your consent at the bottom of this page, you will be reminded of the types of “Grand Challenge” suggestions we are looking for (the same information as in the email), and given an opportunity to input your ideas. You will be asked a few questions about your background. Once you’ve thought of your ideas, the survey should take no more than 10 minutes to complete. All information you provide will be confidential, and you can leave the survey at any point with no consequences.

**Are there any benefits to my taking part?** – Submitting ideas gives you a chance to inform the direction and focus of the RES agenda and activities in the coming years. All suggestions will be given full consideration during an expert-led prioritisation process. Later on, in the prioritisation process (details to follow by email later), you will have the opportunity to volunteer to participate in Zoom meetings, in which submitted suggested will be discussed and prioritised. If you are chosen to participate in these meetings, you will be invited to be a co-author on certain manuscripts and reports that result from the prioritisation process. All members, editors and editorial board members, and associates will be kept informed of the progress of the project through email and/or RES newsletter updates.

**Will my participation be confidential?** Yes. All information collected will remain strictly confidential. Any personal details will be kept in a password-protected file accessible only to the immediate research team. The personal data gathered in this survey will only be used by the immediate research team to assess the diversity of input that we have received and will be deleted before or during February 2021. Although your priority topic suggestions will be used in subsequent prioritisation stages, and may appear in reports and publications, these will not be associated with any of your personal data, and no personal data will appear in reports or publications. General guidance on how the University uses personal data can be found at<https://www.information-compliance.admin.cam.ac.uk/data-protection/research-participant-data>.

**What happens if I change my mind?** - Taking part is entirely voluntary, and refusal or withdrawal will involve no penalty or loss, either now or at any point in the future. You are free to leave the survey at any point or to contact the research team to withdraw your consent at any point in the future. However, after 1st October 2020, your suggestions will have been incorporated in the prioritisation process, and so it would be difficult to withdraw them without affecting the process, but we will consider this, under your instruction, if there is a case to do so.

This research is funded by the Royal Entomological Society (RES), and this project has been approved by the Psychology Research Ethics Committee of the University of Cambridge. Researcher: Dr Sarah Luke (shl47@cam.ac.uk). Research leader: Dr Lynn Dicks (lvd22@cam.ac.uk).

Please click below to acknowledge that you have read, understood, and agreed to the following statements:

□ I confirm that I have read and understood the above Participant Information

□ I understand that I can contact the research team via shl47@cam.ac.uk at any point to ask for more information.

□ I understand that all personal information will remain confidential and that all efforts will be made to ensure I cannot be identified (except as might be required by law).

□ I agree that data gathered in this study may be stored anonymously and securely and will be used later in this “Grand Challenges” prioritisation process.

□ I understand that my participation is voluntary and that I am free to withdraw at any time without giving a reason, up until 1st October 2020.

□ I agree to take part in this survey.

Please provide a contact email address below. This is so that we can: (a) remove your responses at a later point if you choose to do so; and (b) avoid asking you similar questions again when we invite you to participate in later rounds of the prioritisation process.

………………………………………………….

***Third page***

**Instructions for submitting “Grand Challenge” priority topic suggestions**

**Please suggest up to five priority topics on which you think entomologists should focus their efforts over the coming years and decades.**

Think about how you see the future of entomology. What should entomologists be concentrating their efforts on? What can entomology achieve?

**Here are some possible themes to get you thinking, but please let us know what you think and don’t feel limited to these:**

• ‘Blue skies’ science, to better understand the world we live in

• Insects as inspiration for engineering and technological innovation

• The role of entomology in understanding and addressing societal challenges, such as climate change, biodiversity loss, human health

• Knowledge exchange, education, and developing understanding and awareness among scientists, practitioners, and public

• Current practical limitations, skills deficits, and constraints that are holding us back

**Priority topics can be in the form of research questions, questions about the state of knowledge, or statements about problems that need to be overcome. However, please try to be specific enough for people to design a programme of activities or a research agenda. Here are some examples:**

Example 1

• How important is climate change as a driver of insect decline on tropical mountains? JUST RIGHT

• What is driving insect decline and how do we stop it? TOO BROAD!

• Is a change of temperature in the New Forest leading to the loss of the species X? TOO SPECIFIC!

Example 2

• What do we still need to understand about dipteran flight in order to inform the development of micro-aerial vehicles? JUST RIGHT

• What do we need to understand about insect flight in order to inform vehicle design? TOO BROAD!

• What do we need to understand about the function of muscle X in the flight of fly species Y in order to reproduce this in vehicle Z? TOO SPECIFIC!

Example 3

• Limited funding for taxonomic training. JUST RIGHT

• Not enough taxonomic research. TOO BROAD!

• Not enough taxonomists working on family X. TOO SPECIFIC!

Priority topic suggestions should be typed in the boxes below. Each can be up to 280 characters long, and you can submit a maximum of five suggestions.

Suggestion 1 ……………………………..

Suggestion 2 ……………………………..

Suggestion 3 ……………………………..

Suggestion 4 ……………………………..

Suggestion 5 ……………………………..

***Fourth page***

**Participant details**

Question information will be used to assess the range of members who have contributed to ensure that we are hearing from a diverse set of voices.

What is your involvement with the Royal Entomological Society? Please tick all that apply.

□ Fellow

□ Honorary fellow

□ Member

□ Student member

□ Journal editor

□ Journal editorial board

□ Special Interest Group Associate

□ Trustee

What is your gender?

□ Male

□ Female

□ Other

□ Prefer not to say

What is your age?

□ 18-24

□ 25-34

□ 35-44

□ 45-54

□ 55-64

□ 65-74

□ 74+

What is your country of residence?

…………………………………………….

Which category best describes your main current area of entomological activity (or main past area of activity, if you are now retired)?

□ University academic

□ Private sector

□ Non-governmental organisation (NGO)

□ Practitioner (including land managers)

□ Policy-maker

□ Amateur entomologist

□ Educator (e.g. schools, public engagement)

□ Other. Please give details

……………………………………………………

Which of the Royal Entomological Society (RES) journals are you most likely to publish in or to read? Please choose at least one, and up to a maximum of three. Please rank your choices in order of preference/relevance, with 1 denoting the highest preference/relevance.

□ Medical and Veterinary Entomology

□ Insect Conservation and Diversity

□ Agricultural and Forest Entomology

□ Ecological Entomology

□ Systematic Entomology

□ Insect Molecular Biology

□ Physiological Entomology

How many years of experience do you have as an entomologist (either amateur or professional)?

□ 0-10 years

□ 10-20 years

□ 20-30 years

□ 30-40 years

□ 40-50 years

□ 50+ years

**Supplementary Materials Appendix 3** - Additional results details

***Involvement and Scope***

***Stage 1 - Gathering suggested challenges***

- There were no significant differences in the gender profile (ꭓ² = <0.001 df = 1, p = 1), age profile (ꭓ² = 5.83, df = 6, p = 0.44) and country of residence profile (ꭓ² = 4.82, df = 10, p = 0.90) between the full RES membership and those who participated in the first survey.
- Ninety-seven respondents were from the university sector, but 28 of these also listed involvement in other sectors, and the entomological roles of a further 88 other respondents included a wide range of sectors.
- The journals ‘Ecological Entomology’, ‘Insect Conservation and Diversity’, and ‘Agricultural and Forest Entomology’ were listed most commonly by respondents as their area of preferred interest (124, 117, and 89 listings, respectively), but all of the RES’s journals were listed by some respondents (Supplementary Materials Figure S3).

***Stage 3 – Prioritising suggested challenges***

- No queries were raised by any respondents regarding amalgamation decisions.
- There were no significant differences in the gender profile (ꭓ² = 0.93, df = 1, p-value = 0.33), age profile (ꭓ² = 7.24, df = 6, p = 0.30) or country of residence profile (ꭓ² = 10.14, df = 10, p = 0.43) between the full RES membership, and those who participated in the second survey.
- Many respondents were from the university sector (63, of which 15 also listed involvement with other sectors), but a further 55 respondents did not list university involvement and represented a range of other sectors.
- The journals ‘Ecological Entomology, ‘Insect Conservation and Diversity’,’ and ‘Agricultural and Forest Entomology’ were listed as most popular by 74, 66 and 51 respondents, respectively.

***Stage 4– Prioritising shortlisted challenges***

- The 37 voting participants of the workshop included a range of different age groupings and membership types.
- As with the earlier online surveys, the largest single group of participants were university academics (21 in total, but with 7 of these also listing involvement with other sectors), and those who had chosen ‘Ecological Entomology’, ‘Insect Conservation and Diversity’, and ‘Agricultural and Forest Entomology’ as their preferred RES journals (27, 24 and 24 listings, respectively), although a range of roles and journal preferences were represented.

***Emerging themes and priority challenges***

- There was a significant positive relationship between the number of survey respondents who had initially suggested a challenge and the likelihood of it being selected for the final list (z value=2.722, p=0.00648). However, there was variability in this, and some of the suggestions formed from the highest number of amalgamations were not selected, whilst a large number of singly suggested topics (36) were also chosen (Supplementary Materials Figure S4).

**Image credits for Figure 2:** All images are from NounProject.com. Taxonomy = Insect by Hopkins; Blue skies = Sky by Abdo; Methods and Techniques = Definition by Transfer Studio; Anthropogenic impacts = Global Warming by Bartama Graphic; Conservation options = Insect Protection by Annette Spithoven; Ecosystem benefits = Pollination by Nithinan Tatah; Technology and resources = Technology by Kamin Ginkaew; Pests = No Pests by Juraj Sedlák; Knowledge access = Knowledge by Sumit Saengthong; Training and collaboration = Training by Adrien Coquet; and Societal engagement = people by TukTuk Design.

**Table S1** - Details of all participants in the prioritisation process, and their roles.

| **Name** | **Main current area of entomological activity** | **Steering group** | **Processing suggested challenges (Stage 2)** | **Workshop organiser (Stage 4)** | **Voting attendee of workshop (Stage 4)** |
| --- | --- | --- | --- | --- | --- |
| Lynn V. Dicks | University academic | Yes |  | Yes |  |
| Sarah H. Luke | University academic | Yes | Yes | Yes |  |
| Helen E. Roy | University academic, and Non-Governmental Organisation (NGO) | Yes |  | Yes |  |
| Chris D. Thomas | University academic | Yes |  | Yes |  |
| Luke A.N. Tilley | Non-Governmental Organisation (NGO) | Yes |  | Yes |  |
| Simon Ward | Non-Governmental Organisation (NGO) | Yes |  | Yes |  |
| Allan Watt | Research fellow | Yes | Yes | Yes |  |
| Manuela Carnaghi | University academic |  | Yes | Yes |  |
| Maximillian P.T.G. Tercel | University academic |  | Yes | Yes |  |
| Charlie Woodrow | University academic |  | Yes |  |  |
| Sarah L. Barnsley | University academic |  |  | Yes |  |
| Iris Berger | University academic |  |  | Yes |  |
| Miriam Grace | University academic |  |  | Yes |  |
| Coline C. Jaworski | University academic |  |  | Yes |  |
| Eleanor S. Kent | University academic |  |  | Yes |  |
| Francisca Sconce | Outreach & Learning |  |  | Yes |  |
| Natalia B. Zielonka | University academic |  |  | Yes |  |
| Susmita Aown | University academic |  |  |  | Yes |
| Jennifer A. Banfield-Zanin | Private sector |  |  |  | Yes |
| Mark J.F. Brown | University academic |  |  |  | Yes |
| James C. Bull | University academic |  |  |  | Yes |
| Heather Campbell | University academic |  |  |  | Yes |
| Ruth A.B. Carter | University academic |  |  |  | Yes |
| Magda Charalambous | University academic |  |  |  | Yes |
| Lorna J. Cole | University academic |  |  |  | Yes |
| Martin J. Ebejer | Amateur entomologist |  |  |  | Yes |
| Rachel A. Farrow | University academic |  |  |  | Yes |
| Rajendra S. Fartyal | University academic |  |  |  | Yes |
| Fiona Highet | Government entomologist |  |  |  | Yes |
| Jane K. Hill | University academic |  |  |  | Yes |
| Amelia S.C. Hood | University academic |  |  |  | Yes |
| Frank-Thorsten Krell | Museum curator |  |  |  | Yes |
| Simon R. Leather | University academic |  |  |  | Yes |
| Daniel J. Leybourne | University academic |  |  |  | Yes |
| Nick A. Littlewood | University academic |  |  |  | Yes |
| Ashley Lyons | Non-Governmental Organisation (NGO) |  |  |  | Yes |
| Graham Matthews | University academic |  |  |  | Yes |
| Louise Mc Namara | Public Research Agency |  |  |  | Yes |
| Rosa Menéndez | University academic |  |  |  | Yes |
| Peter Merrett | Amateur entomologist |  |  |  | Yes |
| Sajidha Mohammed | University academic |  |  |  | Yes |
| Archie K. Murchie | Research Institute (Government) |  |  |  | Yes |
| Michael Noble | Amateur entomologist |  |  |  | Yes |
| Maria-Rosa Paiva | University academic |  |  |  | Yes |
| Michael J. Pannell | Amateur entomologist |  |  |  | Yes |
| Chooi-Khim Phon | Research Institute (Government) |  |  |  | Yes |
| Gordon Port | University academic |  |  |  | Yes |
| Charlotte Powell | University academic |  |  |  | Yes |
| Stewart Rosell | University academic |  |  |  | Yes |
| Chris Shortall | Private sector |  |  |  | Yes |
| Eleanor M. Slade | University academic |  |  |  | Yes |
| Jamie P. Sutherland | Contract Research Organisation |  |  |  | Yes |
| Jamie C. Weir | University academic |  |  |  | Yes |
| Christopher D. Williams | University academic |  |  |  | Yes |

**Table S2-** The broad ‘Grand Challenge’ theme groupings that emerged from the prioritisation process, and a description of each of the themes. For each theme, subsequent columns show the number of challenges,the number of voters at Stage 3, the threshold number of votes required to progress to Stage 4, and the final number discussed at Stage 4, including wildcards indicated by asterisks. * = 1 challenge with less than the threshold number of votes was recovered as a wildcard. ** = 2 challenges with less than the threshold number of votes were recovered as wildcards.

| **Theme grouping** | **Theme name** | **Theme description** | **Challenges Stage 3** | **Voters Stage 3** | **Votes required to progress to Stage 4** | **Challenges discussed Stage 4** |
| --- | --- | --- | --- | --- | --- | --- |
| Fundamental research | Taxonomy | Taxonomic research, and understanding of what insect diversity exists | 38 | 26 | 2 | 22 |
|  | Blue Skies | Fundamental science research ideas, without an immediate practical application | 60 | 13 | 2 | 20 |
|  | Methods and Techniques | Developing research techniques and methods, to facilitate entomological research | 45 | 12 | 2 | 20** |
| Anthropogenic impacts and conservation | Anthropogenic Impacts | Changes in insect communities, causes of changes | 53 | 34 | 3 | 27 |
|  | Conservation Options | Possible conservation strategies | 36 | 24 | 2 | 25* |
| Uses, ecosystem services, and disservices | Ecosystem Benefits | Benefits we get from insects within ecosystems | 41 | 12 | 1 | 24* |
|  | Technology and Resources | Insects as inspiration for technology, and as a material/resource | 30 | 10 | 1 | 14 |
|  | Pests | Insects as pests: problems and solutions | 55 | 51 | 5 | 36** |
| Collaboration, engagement, and training | Knowledge Access | Access to research resources and knowledge | 29 | 13 | 2 | 12 |
|  | Training and Collaboration | Career development, training, and sharing of ideas, for entomologists | 41 | 19 | 2 | 18* |
|  | Societal Engagement | Engagement of wider society | 44 | 22 | 2 | 24 |


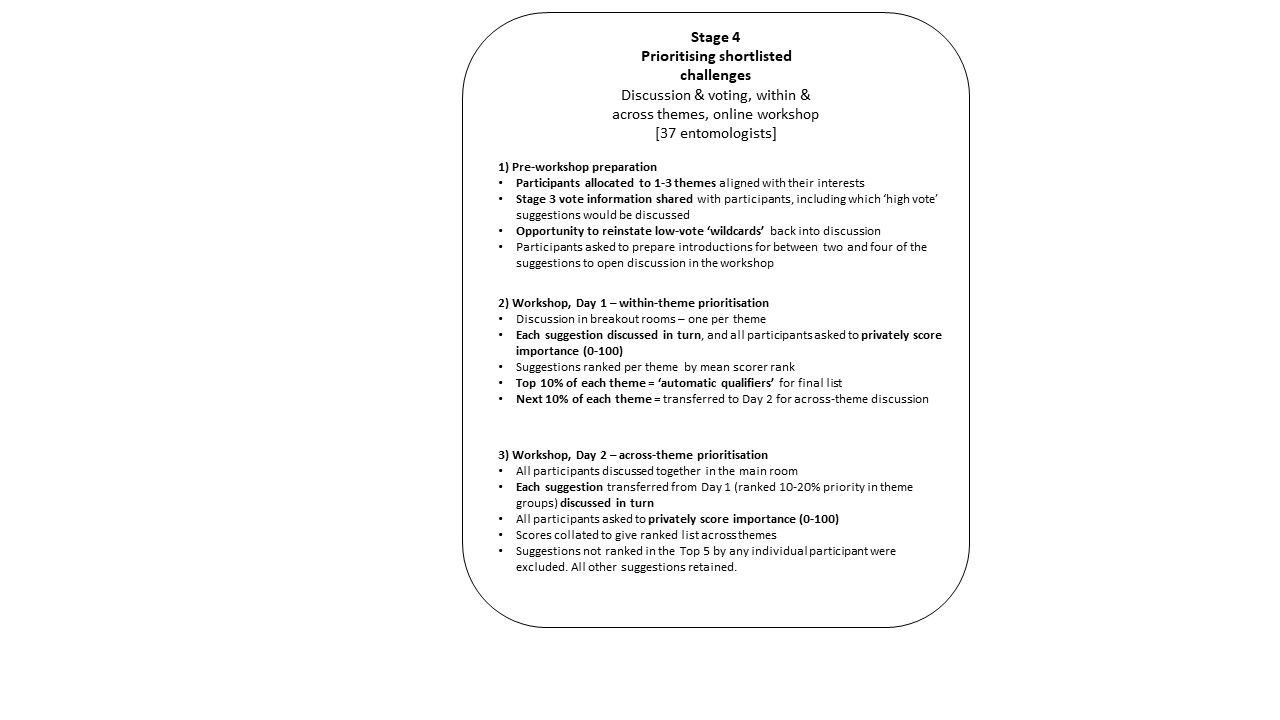


**Figure S1 – Summary of the steps involved within Stage 4 of the collaborative prioritisation exercise.** Stage 4 involved 37 entomologists who were members of the RES and who volunteered to participate in a two-day online workshop involving discussion and voting to determine a priority list shortlist of challenges. The process involved a pre-workshop preparation, within-theme prioritisation, and across-theme prioritisation stages, culminating in production of a final list of suggestions – determined through discussion and voting by participants – by the end of the second day of the workshop. For details of how Stage 4 fits within the wider prioritisation process refer to Figure 1, and details within the main text.


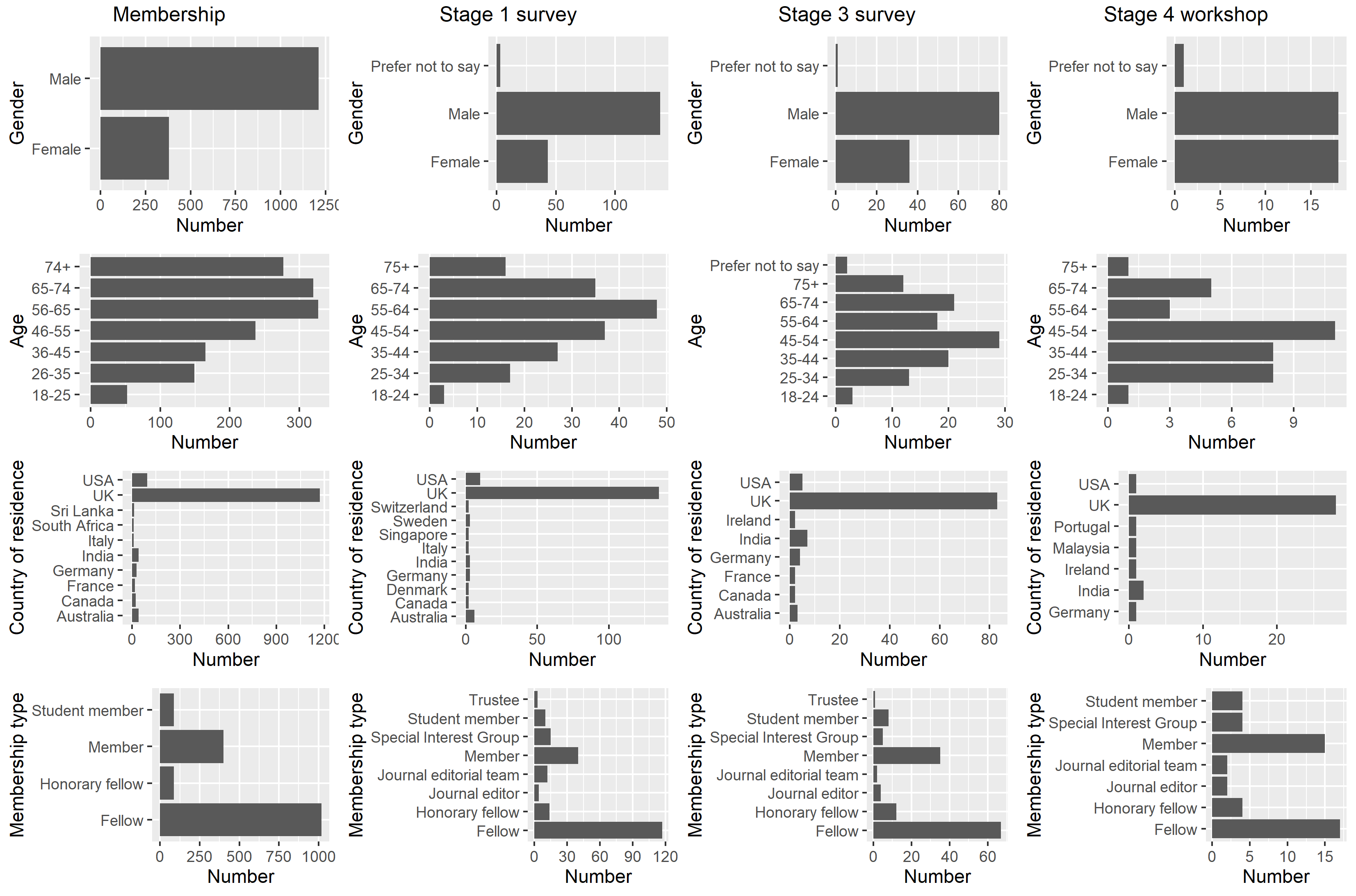
**Figure S2** – Frequency histograms of Gender, Age, Country of Residence, and RES membership type for, from left to right, RES Membership (paying members, excluding journal editorial boards and Special Interest Group members), Stage 1 survey respondents, Stage 3 survey respondents, and voting participants in the Stage 4 workshop. For Membership country of residence, the 10 countries with 10 or more RES members are shown. For Stage 1 and Stage 3, countries with 2 or more respondents are shown. For Stage 4, all countries of residence of participants are shown. Note the shift towards gender balance, mid-career profile and a broader range of membership types, from membership/first survey (similar) to second survey, and then to workshop participants.


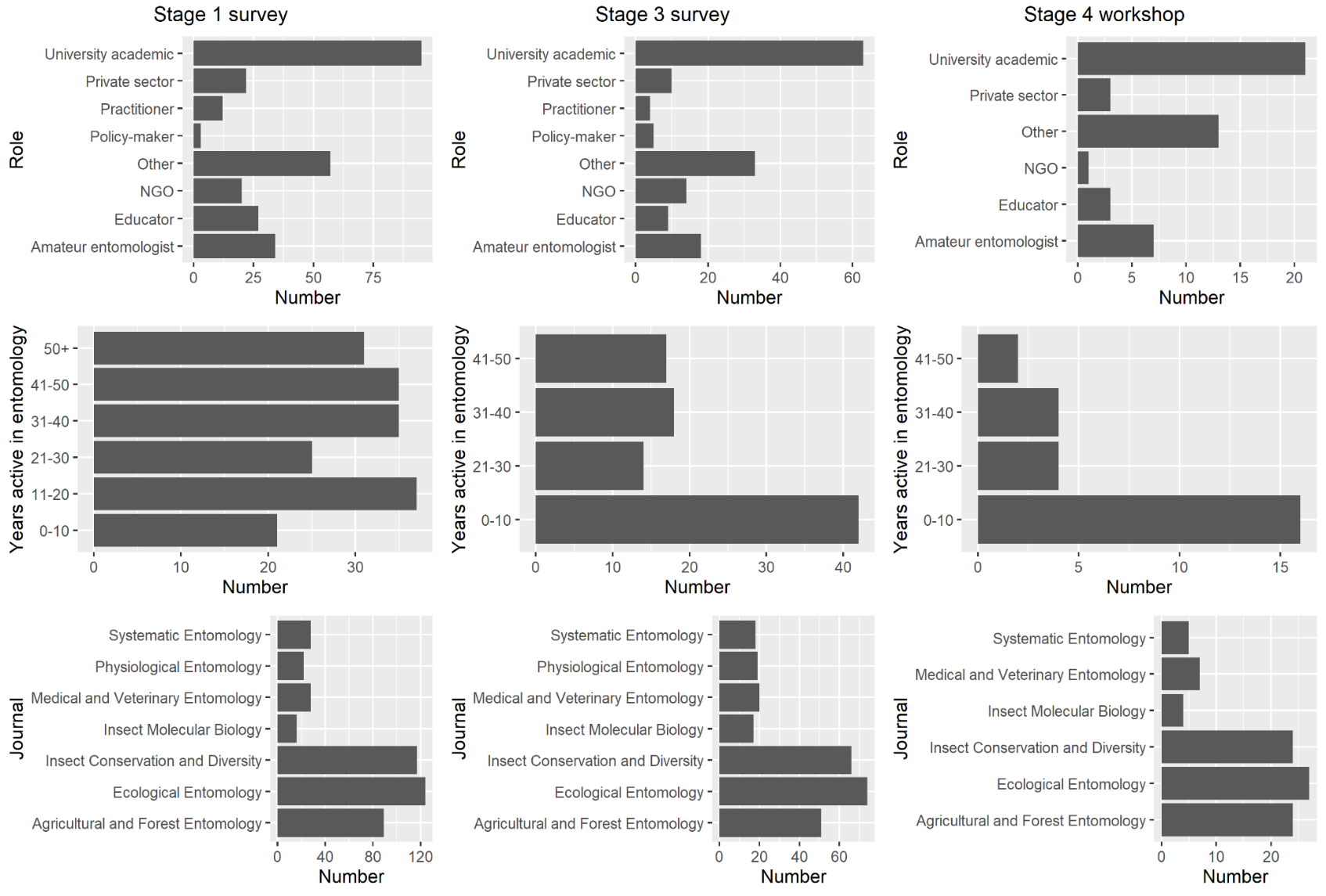
**Figure S3** – Frequency histograms of Role, Age, Years active in entomology, and journal preferences for the respondents to the Stage 1 survey, respondents to the Stage 3 survey, and voting participants in the Stage 4 workshop. These data were not available for the full RES membership. Note the shift towards earlier career stages in the second survey and workshop.


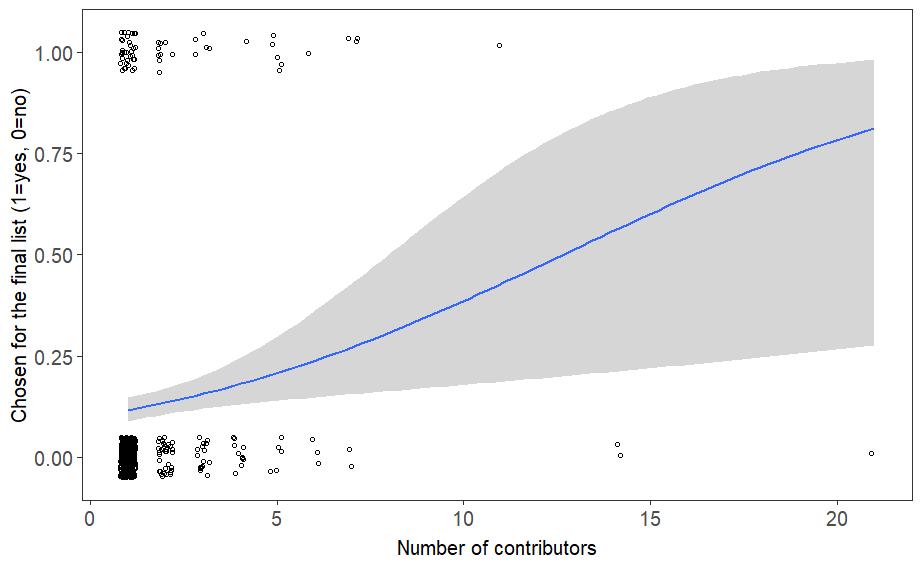


**Figure S4 –** Relationship between the number of contributors who suggested a priority topic in Stage 1 (‘number of contributors’), and whether or not the topic was chosen for the final list of priorities in Stage 4 (‘Chosen for the final list’). Each point represents a suggestion carried through to Stage 3 following amalgamation of duplicates in Stage 2. Location along the x-axis shows how many people suggested that idea in Stage 1, indicating its degree of amalgamation. Location 0 or 1 on the y-axis shows whether it was eventually included in the final list after Stage 4 with 0 indicating ‘no’ and 1 indicating ‘yes’. The curve shows the modelled relationship plus standard error (from a generalised linear model, glm) between the number of contributors and the likelihood of inclusion in the final list (on a continuous scale of 0 to 1, with 0 indicating ‘no’ and 1 indicating ‘yes’).
